# High-Efficiency In Vitro Root Induction in Pear Microshoots (*Pyrus* spp.)

**DOI:** 10.3390/plants13141904

**Published:** 2024-07-10

**Authors:** Jae-Young Song, Jinjoo Bae, Young-Yi Lee, Ji-Won Han, Ye-ji Lee, Sung Hee Nam, Ho-sun Lee, Seok Cheol Kim, Se Hee Kim, Byeong Hyeon Yun

**Affiliations:** 1 National Agrobiodiversity Center, National Institute of Agricultural Sciences, RDA, Suwon 16613, Republic of Korea; jysong77@korea.kr (J.-Y.S.); bjj2021@korea.kr (J.B.); yeji36@korea.kr (Y.-j.L.); creative716@korea.kr (S.H.N.); hosun83@korea.kr (H.-s.L.); sckim12@korea.kr (S.C.K.); 2 Planning & Coordination Division, National Institute of Agricultural Sciences, RDA, Wanju 55365, Republic of Korea; youngyi@korea.kr; 3 Fruit Research Division, National Institute of Horticultural & Herbal Science, Wanju 55365, Republic of Korea; ezsehee@korea.kr (S.H.K.); ybh92@korea.kr (B.H.Y.)

**Keywords:** auxin, IBA, NAA, root induction, plant growth regulator

## Abstract

Extensive research has been conducted on the in vitro mass propagation of pear (*Pyrus* spp.) trees through vegetative propagation, demonstrating high efficiency in shoot multiplication across various pear species. However, the low in vitro rooting rates remain a significant barrier to the practical application and commercialization of mass propagation. This study aims to determine the favorable conditions for inducing root formation in the in vitro microshoots of *Pyrus* genotypes. The base of the microshoots was exposed to a high concentration (2 mg L^−1^) of auxins (a combination of IBA and NAA) for initial root induction at the moment when callus formation begins. The microshoots were then transferred to an R1 medium (1/2 MS with 30 g L^−1^ sucrose without PGRs) to promote root development. This method successfully induced rooting in three European pear varieties, one Asian pear variety, and a European–Asian hybrid, resulting in rooting rates of 66.7%, 87.2%, and 100% for the European pear (*P. communis*), 60% for the Asian pear (*P. pyrifolia*), and 83.3% for the hybrid pear (*P. pyrifolia* × *P. communis*) with an average of 25 days. In contrast, the control group (MS medium) exhibited rooting rates of 0–13.3% after 60 days of culture. These findings will enhance in vitro root induction for various pear varieties and support the mass propagation and acclimatization of pear. The in vitro root induction method developed in this study has the potential for global commercial application in pear cultivation.

## 1. Introduction

The pear (genus *Pyrus*), a member of the *Rosaceae* family, is a widely cultivated temperate fruit crop with significant economic and health benefits across Europe, the United States, and Asia [1,2]. Traditionally, pear propagation and genetic improvement have relied on conventional breeding techniques [3], such as cutting or grafting, which transmit superior genotypes to future generations, ensuring genetic uniformity. However, these methods are limited in extendibility compared to seed propagation, which can produce large numbers of individuals simultaneously. In an effort to overcome the limitations, several studies have reported the use of in vitro culture techniques for the mass multiplication of pear trees via vegetative propagation, aiming to improve regeneration or proliferation rates and plantlet quality [4,5,6,7]. Despite improvements in explant multiplication efficiency in most pear cultivars, a significant challenge remains: the low in vitro rooting rates that impede effective mass multiplication, a common issue in the tissue culture of many woody plants [8].

The in vitro rooting of microcutting explants in *Pyrus* spp. has proven difficult, as reported in several studies [8,9]. Root induction in vitro is significantly affected by various factors including the combinations and concentrations of plant hormones [10], culture medium components, and plant growth regulators (PGRs) [9,10,11,12]. Among these, the response to auxin plays a crucial role in root induction [13]. Rooting induction in *Pyrus* spp. commonly utilizes auxins such as naphthaleneacetic acid (NAA), indole-3-butyric acid (IBA), and indoleacetic acid (IAA) in MS media. NAA is considered the most effective, followed by IBA, IAA, and 2,4-dichlorophenoxyacetic acid (2,4-D) [14], whereas Kaviani et al. [15] reported the most used auxin is IBA rather than NAA. These auxins have been proven successful across various pear cultivars and genotypes, including *P*. *communis*, *P*. *pyrifolia*, and others [16]. Researchers in previous studies have also examined the effects of combining PGRs such as NAA with IBA [7], IBA with cytokines like 6-benzylaminopurine (BAP) [10], or with activated carbon (AC) [17]. However, these methods showed significant differences in rooting frequencies depending on different pear species, or required extended induction periods. Several studies have achieved success in rooting some European pear cultivars but obtained less favorable results with Asian pears, which have harder wood than the European cultivars [8,18,19]. Furthermore, some researchers have noted that there is no single universal method for the in vitro rooting of pear explants due to the variation in genotype response [20,21]. Effective in vitro root induction plays an important role in micropropagation of plant species [22]. Furthermore, the development of rooting systems in vitro is crucial for not only enhancing plant breeding programs and large-scale multiplication but also for the various aspects of plant development and cryopreservation efforts in pear genotypes [7,10]. Although some progress has been made in some pear cultivars, the variability in rooting efficiency across different genotypes necessitates the development of a broadly applicable rooting induction technology [14,23,24].

The present study, hence, was conducted to improve the most suitable medium conditions and establish a broadly applicable method for in vitro root induction by utilizing IBA and NAA as PGRs in European and Asian *Pyrus* germplasm.

## 2. Results

### 2.1. Microshoot Conditions for Root Induction

**Figure 1:**
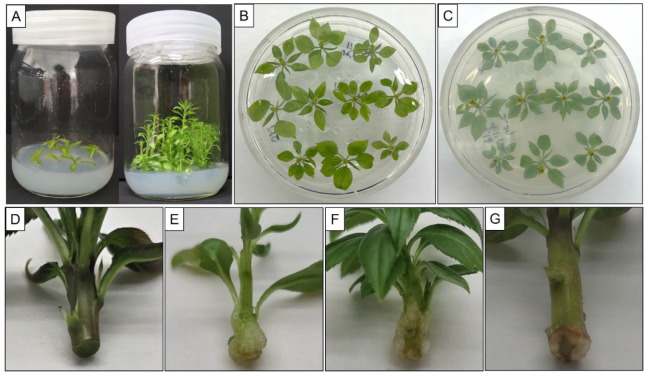
In vitro microshoots for initial root induction. (**A**) In vitro proliferation of microshoot cultures of pear grown on MS medium supplemented with 2 mg L^−1^ BA and 0.2 mg L^−1^ IBA. (**B**) Microshoots on MS medium before transfer to R0-IN medium with auxin. (**C**) Swollen base of microshoots on R0-IN medium. (**D**) Microshoot base before PGR treatment. (**E**) Swollen base of ‘Bartlett’ after 1–2 days on R0-IN medium. (**F**) Swollen base of ‘BaeYun No. 3’ after 10 days on R0-IN medium. (**G**) Swollen base of ‘Oharabeni’ after 3 days on R0-IN medium.

**Figure 2:**
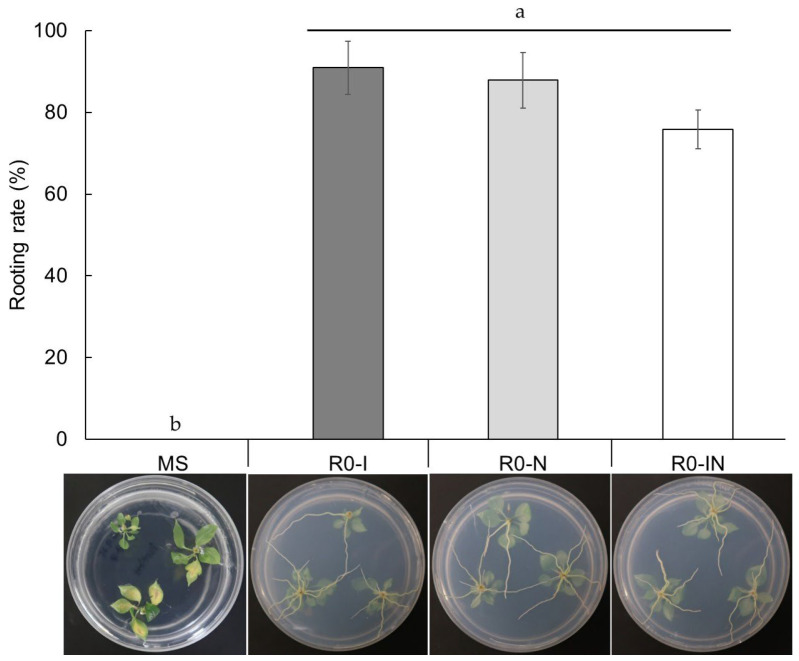
Effect of IBA, NAA, and their combination on the in vitro root formation of ‘Bartlett’ microshoots using a two-step treatment method. MS refers to the rooting rate of microshoots on the MS medium after 30 days of culture. R0-I, R0-N, and R0-IN indicate the rooting rates of microshoots after 1 day of exposure to R0-I (2 mg L^−1^ IBA), R0-N (2 mg L^−1^ NAA), and R0-IN (1 mg L^−1^ each of IBA and NAA), respectively, followed by transfer to the R1 (PGR-free) medium for 14 days. Different letters on bars indicate significant differences at *p* < 0.05 according to Duncan’s multiple range test. Twelve plants per treatment were used, with three replications.

For this study, we used the following plant conditions prior to in vitro root induction: (1) vigorous shoots of six-week-old cultured from shoots (about 1–2 cm) on the propagation medium (Figure 1A); (2) apical microshoots of about 2 cm in length excised from the vigorous shoots (Figure 1B). Considering the age of in vitro shoots, we utilized only apical microshoots from vigorous shoots for in vitro root induction. The physiological age and quality of these shoots were crucial, as poor-quality shoots were insufficient for promoting root formation. The in vitro rooting of microshoots is influenced by various factors, including growth stage, size of explants, subculturing time, PGRs, and vigorous donor material [25,26]. Our results correspond with the studies by Hamad et al. [27] and Tang et al. [28], which emphasized the importance of shoot age in rooting responses.

### 2.2. Initial Exposure Duration to High Concentration of PGRs

To confirm the suitable PGR treatment for root induction, the initial periods of auxin application were tested. We examined the duration before callus formation at the base of the shoot under high auxin exposure (2 mg L^−1^). The duration varied by cultivar, 1–2 days for ‘Bartlett’ (Figure 1E), 10 days for ‘BaeYun No. 3’ (Figure 1F), 3 days for ‘Oharabeni’ (Figure 1G), 2 days for ‘General Leclerc’, and 3 days for ‘Jumbo’ cultivars. The appearance of the swollen bases of microshoots is shown in Figure 1. The type and concentration of auxin are important factors in root induction, but the initial exposure time to high concentrations of auxin is considered critical. Specifically, a key point in this study is the moment when callus formation begins at the base of microshoots. After the specified durations, the microshoots were then transferred to an R1 medium (PGR-free) to promote rooting.

### 2.3. Effect of Different Auxin Types on Rooting Ability

PGRs are generally used for in vitro rooting in many plants. For rooting in *Pyrus*, the most effective and commonly used auxins are IBA [15] and NAA [14]. This experiment focused on the application of IBA and NAA, used alone or in combination, to confirm the efficiency of these PGRs for rooting in a ‘Bartlett’ cultivar. As shown in Figure 2, the rooting rate of microshoots on R0-I moved to the R1 (PGR-free) medium was 90.9%, 87.9% on R0-N to the R1 medium, and 75.8% on R0-IN to the R1 medium after 2 weeks. The results of this investigation for in vitro rooting on different PGR treatments indicated no significant differences in the rooting rate of microshoots among the tested PGRs. These results suggest that it is not the type of hormone that affects rooting efficiency but the timing of transferring the microshoots from a high-concentration auxin treatment to a hormone-free medium that is crucial. The rooting of microshoots in vitro was also examined on the control medium (MS without PGRs), but it was 0% on the MS medium after 30 days, and exhibited a significant difference (*p* < 0.05) in root formation as compared to the tested PGRs.

### 2.4. Effect of In Vitro Rooting Induction Method in Different Genotypes

**Figure 3:**
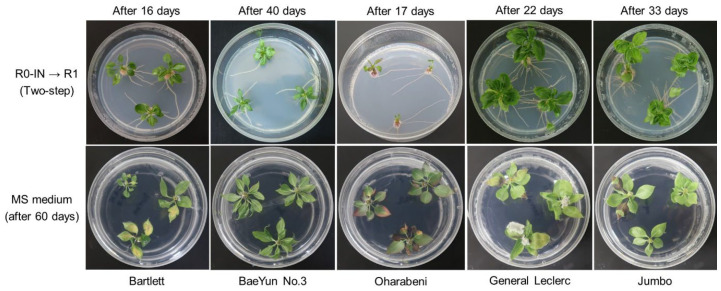
In vitro rooting of five different pear genotypes. The microshoots cultured on the initial root induction medium (R0-IN) in the dark for specified days were moved to the medium (R1).

Our results, although tested with a single variety, revealed no significant difference in rooting rates between the treatments with single auxin or their combination (Figure 2). Previous studies have reported varying effectiveness with different treatments, IBA and NAA combined [8], NAA alone [14], or IBA alone [15]. In this study, five pear cultivars, ‘Bartlett’, ‘BaeYun No. 3’, ‘Oharabeni’, ‘General Leclerc’, and ‘Jumbo’, were subject to rooting experiments. Root induction was performed using the R0-IN medium (PGR combination) in dark conditions for a specified period, followed by the R1 medium in light conditions (Table 1). The rooting rates observed were 100% for ‘General Leclerc’, 87.2% for ‘Bartlett’, and 66.7% for ‘Jumbo’ among the European pear cultivars; 83.3% for the hybrid cultivar ‘Oharabeni’; and 60% for the Asian cultivar ‘BaeYun No. 3’. The Asian pear cultivars generally had more difficulty rooting compared to the European pears. In contrast, the control group (using the MS medium) showed rooting percentages between 0% and 13% after 60 days (Table 1 and Figure 3). These results indicated that the two-step rooting induction method (R0-IN followed by R1) significantly improves rooting rates in all the tested pear cultivars compared to the control (MS medium) (Table 1 and Figure 3). In addition, our results revealed that the in vitro root formation of microshoots could be readily induced, significantly reducing the root formation time to an average of 25 days for five pear genotypes using the in vitro rooting system developed in this study (Table 1). In contrast, previous studies by Liaw et al. [29] and Yi et al. [7] reported that root induction took more than 30 days. Additionally, although the in vitro root induction of pears has been reported, the rooting responses were generally poor [16,30].

## 3. Discussion

The formation of adventitious roots is very difficult to achieve in most pear species [8,9,14]. However, it is crucial for the successful induction of rooting in microshoots obtained from the multiplication stage in most woody plants including *Pyrus* spp. [20,23]. The current study aimed to improve the suitable material and medium conditions, and develop a broadly applicable method for in vitro root induction using IBA and NAA in European and Asian *Pyrus* germplasm.

Thakur et al. [8] reported that high concentrations of auxin induce callus formation at the shoot base, thereby inhibiting normal root growth. We also observed that callus formed at the base of the microshoots when incubated on the medium with high auxin concentrations (2 mg L^−1^). Upon transferring the microshoots with callus to a medium with low concentrations (0.2 mg L^−1^), callus induction continued, but the frequency of roots either slightly increased or exhibited symptoms of withering in our previous study [31]. We found that high concentrations of auxins affected either rooting or callus formation depending on the initial exposure duration. This effect is likely due to increased cell production in the meristem [32], as well as the conversion of differentiated cells at shoot bases into meristematic cells, which can lead to the formation of adventitious root meristems [33,34,35]. Adding auxin to the culture medium can change internal auxin levels, triggering the development of adventitious roots [33]. Our findings suggest that the duration of initial exposure to high auxin concentrations is considered critical, indicating the importance of the point when callus formation initiates at the base of microshoots to facilitate rooting in this study.

Torrey [36] reported that auxins are the main factors in root formation. Some researchers reported the relationship between root formation and auxins, but their opinions differed on the effectiveness of auxins for root induction. The most effective auxin for rooting is reported to be NAA [14], IBA [15,37], and a combination of IBA and NAA [7]. Contrary to these reports, although IBA, NAA, and their combination are effective for rhizogenesis, these results showed no significant differences in rooting rates among these treatments in the ‘Bartlett’ cultivar. The diverse results among various cultivars are mainly due to differences in their indigenous auxin levels [19]. Based on these results, we used a combination of the auxins IBA and NAA to develop a rooting technique applicable to a wide range of pear genetic resources. This method was applied to three European pear varieties, one Asian variety, and a hybrid of European and Asian pears.

The two-step rooting induction method (R0-IN followed by R1), which involves an initial high auxin exposure followed by a PGR-free medium, significantly improves rooting rates in five different pear cultivars compared to the control (MS medium) with 0% rooting. Root formation showed from 60% for the Asian pear ‘BaeYun No. 3’ to 100% for the European pear ‘General Leclerc’ cultivar. The Asian pear cultivars tended to generally have more difficulty rooting compared to the European pears. Similar results were reported for successful rooting in European pears, whereas the results were less favorable with Asian cultivars [8,18]. According to some researchers, in vitro rooting results showed a maximum of 72% in *P. syrica* [17], 14% to 27% in *P. pashia* [38], and 6% to 50% in *P. communis* [9]. The results were poorer, or shoots failed to root, with Asian cultivars [8,9]. In this study, the rooting formation indicated more than 60% for all the tested cultivars including an Asian cultivar. This study successfully established an effective in vitro rooting induction method for *Pyrus* genotypes, significantly enhancing rooting rates and reducing root formation duration.

## 4. Materials and Methods

### 4.1. Plant Material

Five pear cultivars, *P. communis* ‘Bartlett’ (IT 226391), *P. pyrifolia* Nakai ‘BaeYun No. 3’, *P. pyrifolia* × *P. communis* ‘Oharabeni’ (IT 226170), *P. communis* ‘General Leclerc’ (IT 243471), and *P. communis* ‘Jumbo’ (IT 226267), were used as plant material for in vitro root induction. The genotypes were obtained from the National Institute of Horticultural and Herbal Science (NIHHS) of the Rural Development Administration (RDA). The microshoots of about 2 cm in length were utilized for in vitro shoot proliferation and root induction. All the cultures were maintained in a culture room at 24 ± 1 °C, under a 16 h photoperiod, in closed vessels with consistently high humidity.

### 4.2. In Vitro Shoot Proliferation

Shoot cultures were established following the methods previously described by Yi et al. [7]. Briefly, shoots and stem explants from five genotypes were cultured on the Murashige and Skoog (MS) medium [39] supplemented with 2.0 mg L^−1^ N6-benzyladenine (BA) and 0.2 mg L^−1^ indole-3-butyric acid (IBA) containing 30 g L^−1^ sucrose, and adjusted to a pH of 5.8. Vigorously growing shoots were obtained on this medium after 6 weeks of culture. These shoots were subsequently used for the in vitro root induction experiments (Figure 1A).

### 4.3. Application of Different Auxin Types

To investigate the efficiency of PGRs in inducing roots in pear microshoots, we prepared the 1/2 MS medium including different auxins (IBA and NAA) and MS medium as a control. The microshoots were treated with the following media: R0-I (1/2 MS with 2 mg L^−1^ IBA and 20 g L^−1^ sucrose), R0-N (1/2 MS with 2 mg L^−1^ NAA and 20 g L^−1^ sucrose), and R0-IN (1/2 MS with a combination of 1 mg L^−1^ IBA and 1 mg L^−1^ NAA and 20 g L^−1^ sucrose). For each of these media, the microshoots of the ‘Bartlett’ were cultured in the dark for 1 day and then transferred to the R1 medium for root formation using a two-step method (Table 2). The R1 medium contained 1/2 MS with 30 g L^−1^ sucrose without PGRs. The percentage of root formation was monitored after 14 days of treatments, as shown in Figure 2. The percentage of root formation, regardless of the number of roots, was determined by the number of microshoots that formed roots longer than 1 cm relative to the total number of microshoots used in this experiment. For each treatment, twelve plants were employed, and the experiment was replicated three times.

### 4.4. In Vitro Root Induction for Different Gentoypes

In our previous study [31], we developed a method for the in vitro rooting of an ‘Oharabeni’ (*P. pyrifolia* × *P. communis*) cultivar. Briefly, microshoots were initially cultured on a root induction medium (R0) in the dark for three days. Following the initial root induction phase, the microshoots were transferred to five rooting expression media (R1–R5), respectively. In the present study, among these conditions, we selected the R0-IN to R1 transfer as providing the highest in vitro rooting efficiency and conducted further experiments using five different cultivars. Microshoots were cut from the propagated shoots of five cultivars (Figure 1D) and transferred to MS medium for one week (Figure 1B). The microshoots were then cultured on the R0-IN medium in the dark for 1–10 days (Figure 1C), depending on the cultivar, before being transferred to the R1 medium to induce root formation (Table 2). 

### 4.5. Statistical Analysis

Data were analyzed by the analysis of variance (ANOVA) and Duncan’s multiple range test at a significance level of 0.05, utilizing the SAS software (Statistical Analysis System, Version 7.1, Cary, NC, USA). A *t*-test was conducted to compare the statistical differences in the rooting rates between the control (MS medium) and the rooting method (R0-IN→R1) using Microsoft Excel 2016.

## 5. Conclusions

This study describes a method in which microshoots are transferred to a hormone-free medium at the critical point when callus formation begins at the base of the microshoots following treatment with a high concentration of auxins. This critical period varies by cultivar. The method of root induction involved the following procedures: the R0-IN medium (1/2 MS with a combination of 1 mg L^−1^ IBA and 1 mg L^−1^ NAA, and 20 g L^−1^ sucrose) in dark conditions for a specified period, when callus formation begins, followed by the R1 medium (1/2 MS with 30 g L^−1^ sucrose without PGRs) in light conditions. This method resulted in a good rooting response in different pear genotypes. It is important to note the initial treatment (exposure) time. The rooting results obtained in this study confirmed that adventitious root formation in pear requires an initial high concentration of auxin treatment and that the duration of auxin shock determines rooting efficiency. The in vitro rhizogenesis method developed in this study showed high rooting rates for European pear (*P. communis*, 66.7%, 87.2%, and 100%), Asian pear (*P. pyrifolia*, 60%), and a hybrid of pear (*P. pyrifolia* × *P. communis*, 83.3%). Therefore, this result suggests that the protocol described in this study could be widely used as an effective method for the in vitro root induction of *Pyrus* germplasm and for other woody plants. Further studies are underway to test the suitability of the method used in this study for other pear genotypes.

## Figures and Tables

**Table 1:** Results of the application of rooting induction technology in five pear cultivars.

Cultivars	Rooting Rate (%)		R0-IN Medium in Dark Condition (Days of Treatment)	R1 Medium in Light Condition (Days of Treatment)
	MS Medium (after 60 Days)	R0-IN→R1 Medium		
Bartlett	0.0	87.2 ± 6.0 ab ***	1 or 2	14
BaeYun No. 3	0.0	60 ± 12.5 b **	10	30
Oharabeni	13.3 ± 8.2	83.3 ± 8.3 ab **	3	14
General Leclerc	13.3 ± 8.2	100 a ***	2	20
Jumbo	0.0	66.7 ± 14.5 b **	3	30

R0-IN medium (1/2MS + 20 g L^−1^ sucrose + 1.0 mg L^−1^ IBA + 1.0 mg L^−1^ NAA). R1 medium (1/2MS + Sucrose 30 g L^−1^ without PGRs). A *t*-test for comparisons of statistical differences in the rooting rates between the control (MS) and the rooting method (R0-IN→R1) for each cultivar. *T*-test was applied (** *p* < 0.01, and *** *p* < 0.001). Data with different letters in a column indicate significant differences at *p* < 0.05 according to Duncan’s multiple range test. Fifteen microshoots were utilized for each treatment, with three replications.

**Table 2:** Composition of media used for in vitro root induction in pear cultivars in this study.

Rooting Media	1/2 MS (g L^−1^)	Sucrose (g L^−1^)	IBA (mg L^−1^)	NAA (mg L^−1^)	pH	Plant Agar (g L^−1^)
R0-IN	2.2	20	1	1	5.8	8
R0-I	2.2	20	2	-		
R0-N	2.2	20	-	2		
R1	2.2	30	-	-		

R0-IN, R0-I, and R0-N, culture medium for initial root induction, cultured in the dark for specified days and transferred to the R1 medium.

## Data Availability

Data are contained within the article.
